# Evaluating an urban pediatric hospital’s scald burn prevention program

**DOI:** 10.1186/s40621-021-00314-0

**Published:** 2021-09-13

**Authors:** Rochelle Thompson, Ross Budziszewski, Autumn D. Nanassy, Loreen K. Meyer, Paul Glat, Brooke Burkey

**Affiliations:** 1 St. Christopher’s Hospital for Children, Philadelphia, PA 19134 USA; 2 Drexel University College of Medicine, Philadelphia, PA 19129 USA

**Keywords:** Prevention, Caregiver education, Scald burns, Hospital programming

## Abstract

**Background:**

Over 450,000 individuals are hospitalized with burns annually and roughly 35% are scald burns. Children younger than 5 years of age are at the greatest risk of scald burn injury. Caregiver burn prevention programs have been found to reduce the prevalence of injuries in young children; however, low-income and underserved populations seldomly have access to these programs. The impact of scald burn prevention programs in underserved populations remains unexplored. The objective of the current study was to evaluate the efficacy of a scald burn prevention program at a Level One Pediatric Trauma Center in a low-income, underserved community.

**Methods:**

Our hospital developed a one-hour scald burn prevention program for caregivers with children 5 years of age or younger. The program educated caregivers on ways to prevent scald burns and create safeguards in their home. Caregivers completed a pre-post survey to measure their ability to identify hot or cold objects, as well as respond to items about their perceptions of the program’s utility, their willingness to share it with others, and the likelihood that they would use the information in the future. Data was analyzed using a paired *t*-test.

**Results:**

Two-hundred and sixty-nine (*N* = 269) caregivers participated in the program. Before the program, caregivers could identify potentially hot objects 83.17% of the time, and after the program, they were able to identify these items 92.31% of the time: *t* (268) = 12.46, *p* < .001, *d* = 1.07. Additionally, 95% of caregivers indicated that the program was helpful, 99% stated that they were likely to share this information with others, and 100% indicated that they would use the information from the program.

**Conclusions:**

Education is a critical component to prevent scald burns. Results indicate that a hospital-led scald burn prevention program can positively impact a caregiver’s ability to identify possible scald-burn risks. Providing education to caregivers who typically do not receive this information could lower the prevalence of scald burns not only institutionally, but in communities that are disproportionately impacted by this mechanism of injury.

## Background

According to the American Burn Association (ABA), approximately 450,000 individuals are hospitalized from burns annually (American Burn Association 2020). Of these hospitalized injuries, scald burns account for 35% of burns. Scald burns disproportionately impact children, with 60% of scalds occurring in children 5 years and younger (UC San Diego Health 2020; Krishnamoorthy et al. 2012; Jeschke et al. 2020). Scald burns can be caused by contact with hot liquid or steam and often occur in kitchens or while children are being bathed (World Health Organization 2020). Young children who experience scald burns can experience life-long physical and psychosocial problems ranging from post-traumatic stress disorder (PTSD) to loss of function of an area of their body (American Burn Association 2020; Stoddard et al. 2017; Sharma and Parashar 2010). Children who grow up in areas with less access to healthcare and come from low-income families are more likely to suffer longitudinal impacts from scald burns (Patel et al. 2018). Because of the high prevalence and myriad of problems that scald burns can create for young children from low-income areas, preventing future burn injuries in this population is imperative.

Previous research shows that community-based prevention programs can reduce the occurrence of scald burns in young children by educating caregivers on how to create safer homes (Turner et al. 2004; Cagle et al. 2006). As such, Cagle and colleagues reported that caregivers who participated in workshops at local community centers were 60% more likely to purchase and install antiscald devices in their kitchens (Cagle et al. 2006). Other programs have utilized mobile applications to provide caregivers knowledge regarding scald burns, specifically, assisting individuals with how to identify possible hot surfaces and found that showing illustrations of hot surfaces was effective to increase caregiver knowledge (Burgess et al. 2018). While community-based programs are useful, they are often difficult for individuals who reside in lower socioeconomic areas to access due to lack of technology, financial flexibility, transportation, or low reading levels. (Adler and Newman 2002; Barlow et al. 2017). Therefore, existing community-based programs may not be sufficient to reach residents in low socioeconomic areas.

Hospital-based programs are useful to help provide caregivers with prevention education while they bring their child in for medical services. Specifically, research has found that hospital-based prevention initiatives are not only a part of holistic patient care but are perceived as more accessible for low-income patients with many individuals reporting that they are more willing to attend if they are in a hospital rather than a community setting (Gold et al. 2018; Peck et al. 2016). While hospital prevention programs may limit the barriers that exist in community programs, making program information easy to understand is essential for program success. Parbhoo and colleagues found that providing parents with pictorial representations of hot items was more impactful than written text (Parboo et al. 2009). Similarly, it has been noted in the past that multiple methods of education (e.g., lecture and hands-on activities) are more impactful than just a single method of learning (Miller-Day et al. 2013).

The present study aimed to utilize prior research to design a program targeting scald burn prevention in a low-income community. We provided a hospital-based scald burn prevention program that utilized lecture and hands-on demonstrations. This program took the hospital’s population into account and was specifically tailored to be culturally relevant and appropriate for all caregiver reading levels. The goal of the current study was to understand the efficacy of the program in increasing the overall scald burn knowledge of caregivers of children 5 years of age or younger.

## Methods

### Program setting

The program was developed and implemented at a 188-bed, urban, Level I Pediatric Trauma Center in the Northeast region of the United States. The hospital is verified by the ABA as a Pediatric Burn Center and serves a high volume of pediatric burn patients annually (St. Christopher’s Hospital for Children 2020). The emergency department receives over 70,000 patients yearly and is one of the busiest in the country (The Philadelphia Inquirer 2020). The hospital is also located in one of the poorest congressional districts in the United States, with 50% of children residing in households at or below the poverty line (Food Research and Action Center 2020).

### Program description and measures

The current program was acknowledged by our Institutional Review Board (#2004007794). The hospital’s Injury Prevention and Research Coordinators developed a one-hour scald burn prevention program that invited caregivers who had children 5 years or younger to participate from 2018 to 2019. Caregivers were referred to the program by various hospital staff members such as social workers, physicians, nursing staff, and the Injury Prevention Coordinator. Caregivers that attended the hospital-based program were from a variety of locations, including in-patient and out-patient facilities, community referrals, and individuals with children presenting to the hospital’s Emergency Department. Caregivers were invited to the scald burn prevention course held at the hospital. Each session had 5–10 caregivers in attendance to ensure caregivers were able to receive one-on-one attention from the instructor, feel comfortable asking questions, and participate in hands-on activities.

**Fig. 1:**
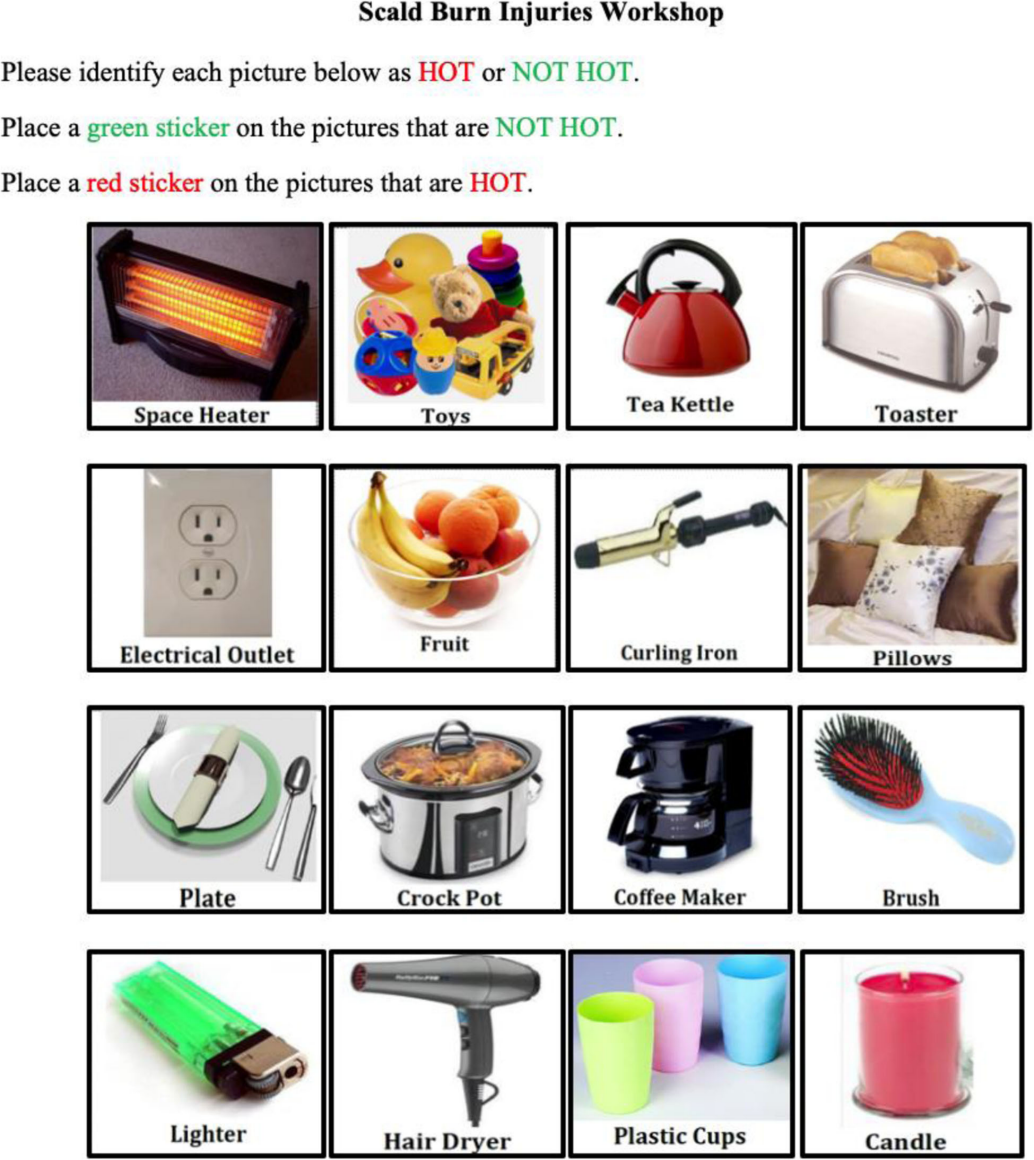
‘Hot or Not’ Activity Used For Pre-Post Knowledge Assessment. Figure Note. Caregivers were asked to identify items that could possibly be hot at any time. Hot items were as follows: Space Heater, Tea Kettle, Toaster, Electrical Outlet, Curling Iron, Plate, Crock Pot, Coffee Maker, Lighter, Hair Drier, and Candle

The course began with a pre-test survey asking the caregivers to identify 16-items as ‘hot or not’ (See Fig. 1). In this survey, caregivers were asked to place a green sticker over items that were ‘NOT HOT’ and a red sticker over pictures that were ‘HOT.’ Then, the Injury Prevention Coordinator gave a 45-min instructional and demonstration lecture on the dangers of scald burns, common scenarios where scalds occur, and prevention tips for the caregivers. A special emphasis was placed on identifying hot items and creating kitchen safeguards, ‘NO KID ZONES,’ in their homes to keep children away from areas of possible scald burn risks. Following the course, caregivers were provided with an opportunity to use appliances and other burn-prevention equipment (i.e., cabinet locks, travel mugs, stove-covers, safety bath ducks) to understand how they function.

At the end of the course, a post-test survey was distributed with the same 16-items as the pre-survey, but also included three questions asking about: (1) the utility of the program, (2) willingness to share the program’s information with others, and (3) if they will use the information they learned. For completing the program, caregivers were provided with prevention items such as temperature safety ducks to assist parents to test water, reading packets, no-spill travel mugs, infant bathtubs, plug-ins, cabinet locks, suction bowls and child safety gates to create safer home environments. These items can be useful in preventing common scald burns and were introduced with descriptions of appropriate use in the demonstration from the injury prevention coordinator.

### Data analysis

Data from the paper survey was entered into Microsoft Excel (Seattle, Washington, USA) and composite scores were compared from pre-post. The post survey items regarding the utility, willingness to share the information, and likelihood to use the program’s content were represented by group mean scores (in percentages). A paired *t*-test was utilized to display group differences from pre to post survey on the ‘hot or not’ activity. Effect size (Cohen’s *d*) was calculated to identify the magnitude of the change. Statistical analyses were performed using R Studio (R Studio Team 2020).

## Results

Over the two-year program, 269 caregivers participated and over 700 prevention items were distributed. Before the program, caregivers were able to correctly identify 13.31 hot objects out of 16 (*SD* = 1.66; 83.17%). After the program, caregivers were able to identify 14.77 (*SD* = 0.99; 92.31%). The difference in mean scores from pre to post was statistically significant: *t* (268) = 12.46, *p* < .001, *d* = 1.07. Additionally, 95% of caregivers indicated that the program was helpful, 99% stated that they were likely to share this information with others, and 100% indicated that they would use the information from the program.

## Discussion

Results displayed that the one-hour prevention course significantly increased caregiver knowledge and that caregivers found the program useful and were likely to share the information with others. Moreover, results emphasized that high mean scores in the post-program group were heavily influenced by the program content, explaining the large effect size (Lakens 2013). While providing a scald burn prevention program was not novel by itself, the integration of a low-cost, brief, and easy to understand program that caregivers can receive while receiving care for their child at the hospital provides evidence that outreach efforts do not need to be separate from patient care at a hospital. As previously described, scald burn prevention programs have been successful to promote installation of antiscald kitchen appliances or mobile applications; however, these programs required caregivers to purchase these tools or have a phone capable of downloading the application which limited the programs’ reach (Cagle et al. 2006; Burgess et al. 2018). The present study provided all materials that were introduced such as water-thermometers, travel mugs, and stove knob covers that are low-cost solutions that require no technology and were of no cost to the caregiver. While it may be difficult to establish funding across hospitals, it should be noted that these simple prevention items can come at low-costs and may eliminate perceived barriers in prevention programs.

The success of the current program may also be due to the simplicity of the programming and associated activities. Rather than providing a technology-based education program or reading information, the current program utilized lecture and hands-on demonstrations as the main modes of learning. It has been shown that prevention programs that use multiple methods of education rather than relying on caregivers reading or listening to lectures increases knowledge at a greater rate (Giuse et al. 2012; Vogl et al. 2012). The present program illustrates that demonstrating proper use of prevention materials in real-life settings before providing these items to caregivers increases the caregiver’s perception of their usefulness (Miller-Day et al. 2013). Therefore, prevention programs should make an effort to provide programming that is interactive and demonstrates usage of scald burn prevention tools that will mirror what the caregiver will use in their home.

Additionally, the evaluation tool, while simplistic, ensured that participants were able to understand the directions and apply information from the class into a practical assessment. As such, the current study used pictorial representations of items that would be discussed during the program that caregivers and their children encounter on a daily basis. Pinalla et al. found that appropriate evaluation of programming should not include situations that are unlikely to occur in real life (Pinilla et al. 2014). Related to this, Parbhoo and colleagues noted that when surveys are written, certain populations, especially in low-income areas, may have a low effectiveness to measure learning outcomes due to potential misunderstandings (Parboo et al. 2009). Thus, findings from this study shed light on the need to make sure that during program evaluations surveys need to utilize pictorial items or make sure that the reading level is suitable to the population of interest.

Lastly, the current program aimed to decrease perceived barriers to access programs by providing caregivers access to educational resources while they were at the hospital. By providing programing at hospitals, caregivers did not need to find transportation to and from the program. Rather, they were able to participate while their child(ren) received standard medical care or were being treated for their burn. While scald burn programs are often offered at community centers, there are often transportation and/or childcare barriers, which may limit attendance (Hodkinson et al. 2017). Additionally, many areas of low-socioeconomic status do not have educational resources readily available (Silverman et al. 2016). Therefore, when hospitals are located in these areas, they should provide these programs to really emphasize holistic patient care.

### Limitations and future directions

The current study is not without limitations. Most notably, due to this being a quality assurance project, the author’s Institutional Review Board prohibited the collection of identifying information beyond initials to link pre-post responses. This limited the present study’s ability to accurately depict the sample; however, the general geographical location and hospital’s surroundings were included to provide some background of the population that is usually served. First, the current study displayed that a hospital-led program could increase caregiver knowledge in a generally low-income community, yet it did not show if the program reduced rates of scald burns in the hospital’s patient population. This was hospital-based at a single center; thus, this program should be replicated at other facilities to understand if it is generalizable. The current project utilized the same, unvalidated, survey for English and non-English speakers, however, due to lack of resources to translate the other survey questions, data was not able to be included for this population in the present sample. Lastly, participants could have perhaps stated that the program was more helpful than they actually found it due to providing socially desirable answers. Qualitative feedback could perhaps reduce these confounding factors and specifically understand what was helpful for each participant.

The results from the current study expand literature and could inform future research. As such, future work should investigate if the implementation of scald burns programs at a hospital decrease scald burn occurrences longitudinally at an institution. Along similar lines, longitudinal designs would allow for researchers to test the durability and long-term improvement of knowledge gained from the program. Additionally, understanding and following-up with caregivers to assure that they were able to implement the tools that were distributed in their home would be helpful to understand if the program truly taught them how to utilize these resources. Moreover, future work could attempt to create a validated knowledge assessment to specifically target scald burn knowledge items and assure their validity. Further, future research should try to establish if the program is impactful to Spanish or non-English speaking caregivers, even just through instructor demonstrations. Lastly, future research should attempt to garner community partnerships to have interconnected prevention programs that offer the same information at hospital and community settings.

## Conclusions

The present study found that a one-hour hospital-led scald burn prevention course significantly increased caregiver knowledge and that caregivers found the program useful. Moreover, caregivers reported that they were extremely likely to use the materials they received and that they planned to share this information with others. The results from the current study show that a simple scald burn program can assist caregivers, regardless of their background, in understanding potential scald burn risks while providing useful materials to attempt to prevent future burns.

## Data Availability

The datasets used and analyzed during the current study are available from the corresponding author on reasonable request.

## Abbreviations

ABA: American Burn Association
PTSD: Post traumatic stress disorder
